# Phenotype Switching in Melanoma: Implications for Progression and Therapy

**DOI:** 10.3389/fonc.2015.00031

**Published:** 2015-02-13

**Authors:** Frederic Zhentao Li, Amardeep Singh Dhillon, Robin L. Anderson, Grant McArthur, Petranel T. Ferrao

**Affiliations:** ^1^Oncogenic Signaling and Growth Control Program, Research Division, Peter MacCallum Cancer Center, East Melbourne, VIC, Australia; ^2^Sir Peter MacCallum Department of Oncology, The University of Melbourne, East Melbourne, VIC, Australia; ^3^Department of Pathology, The University of Melbourne, East Melbourne, VIC, Australia; ^4^Metastasis Research Laboratory, Research Division, Peter MacCallum Cancer Center, East Melbourne, VIC, Australia; ^5^Department of Medicine, St Vincent’s Hospital, The University of Melbourne, East Melbourne, VIC, Australia

**Keywords:** melanoma, phenotype switching, EMT, metastasis, RTK signaling, BRAF inhibition, resistance

## Abstract

Epithelial–mesenchymal transition (EMT) is a key process associated with the progression of epithelial cancers to metastatic disease. In melanoma, a similar process of phenotype switching has been reported and EMT-related genes have been implicated in promotion to a metastatic state. This review examines recent research on the role of signaling pathways and transcription factors regulating EMT-like processes in melanoma and their association with response to therapy in patients, especially response to BRAF inhibition, which is initially effective but limited by development of resistance and subsequent progression. We highlight studies implicating specific roles of various receptor tyrosine kinases (RTKs) in advancing melanoma progression by conferring a proliferative advantage and through promoting invasive phenotypes and metastasis. We also review the current knowledge of the mechanisms underlying resistance to BRAF inhibition and the potential role of melanoma phenotype switching in this process. In particular, we discuss how these important new insights may significantly enhance our ability to predict patterns of melanoma progression during treatment, and may facilitate rational development of combination therapies in the future.

## Introduction

Malignant melanoma accounts for 75% of deaths from all skin cancers in the U.S (1). Women have higher survival than men (2) and the Caucasian population has a 10-fold greater risk than ethnic groups with deeply pigmented skin (3). The 5-year survival rate is over 90% for localized melanoma but drops to 16% for distant-stage disease (1), indicating that metastasis is the main reason for poor outcome. The classic Clark model depicts step-wise transformation of melanocytes to malignant melanoma and subsequent development of invasion and metastasis (4), involving tightly regulated switching of cellular phenotypes. This phenotype switch bears resemblance to the epithelial–mesenchymal transition (EMT), a well-characterized process of phenotypic change that is associated with metastatic progression in epithelial cancers. This mini-review will focus mainly on the signaling and molecular events that lead to the invasive and metastatic phenotypes of melanoma, and discuss the implications of phenotype switching on the response to treatment.

## Characteristics of EMT in Epithelial Cancers

Epithelial–mesenchymal transition has been suggested to play an important role in conferring metastatic properties in many solid tumors by altering the integrity of cell–cell junctions, promoting loss of polarity and epithelial markers, eventually resulting in loss of contact between neighboring cells. Through this process, tumor cells become more mesenchymal-like, exhibiting higher migratory and invasive properties that allow them to interact with the extracellular matrix and invade surrounding tissues (5). It is generally accepted that the EMT process involves changes in expression of epithelial and mesenchymal markers. The loss of E-cadherin is a characteristic feature during EMT, which is detected in the cells located at the invasive front of many solid tumors (6, 7). The expression of E-cadherin is tightly regulated by multiple transcription factors that bind to and repress the activity of the E-cadherin promoter (8, 9). The first characterized direct repressor of E-cadherin was the zinc-finger transcription factor Snail1 (10, 11), which initiated intense efforts to understand the molecular mechanisms of EMT and subsequently led to the discovery of the E-cadherin repressors SNAI2 (also known as SLUG) (12), ZEB1 and ZEB2 (13, 14). Other repressors of E-cadherin include E47 (TCF3), TCF4 (15), and Twist (16), which participate in both developmental EMT and tumor progression. Beta-catenin/TCF4 binds directly to the ZEB1 promoter and activates its transcription, conferring invasiveness in colorectal cancer (17).

A common signaling mechanism that induces EMT in a range of cancers is activation of the MAPK/ERK pathway, which can activate SNAI1 to repress E-cadherin expression and the epithelial phenotype (18). In addition, EGF signaling can induce TWIST through a JAK/STAT3 pathway in epithelial cancer cell lines and the EGF–STAT3-positive correlation has been confirmed in primary breast carcinomas (19). Receptor tyrosine kinases (RTK) signaling activated through FGF, HGF, IGF, and other ligands, as well as the serine/threonine kinase TGF-β superfamily, can also initiate EMT and metastasis, through various mechanisms converging on the induction of E-cadherin repressors (20, 21).

## Evidence of EMT-Like Phenotype Switching in Melanocytes and Melanoma

Epithelial–mesenchymal transition is a critical step for embryonic morphogenesis and a similar process is particularly important for melanocyte lineage differentiation. It involves restructuring of the cytoskeleton, cell membrane, and cell–cell junctions. This developmental plasticity allows melanocytes to emerge from the pluripotent neural crest cells (22). Phenotype switching with similarities to the EMT program operates during development and has a recognized role in acquisition of metastatic properties in the vertical growth phase of melanoma (23). A comparison of the features of primary cutaneous melanomas from the patients who develop metastasis to those who do not, revealed differences in the expression of the epithelial and mesenchymal phenotype markers (24). By gene expression profiling, loss of E-cadherin with gain of N-cadherin and osteonectin (SPARC) was significantly associated with development of metastasis (24). Further evidence comes from the finding that both proliferative and invasive cells are present within heterogeneous metastatic tumors, and the observation of switching between the two phenotypes during melanoma progression *in vivo* (25).

## Inducers of EMT-Like Phenotype Switching in Melanoma

Recently, the concept of an EMT spectrum has been introduced to describe a progressive transition characterized by an intermediate mesenchymal status and fluctuating expression of EMT markers, as reported in carcinomas of the breast, colon, and ovary (26). Given the intermediate mesenchymal nature of melanoma, fluctuating expression of EMT inducers are observed. Therefore, the literature about phenotype switching in melanoma and about EMT in many epithelial cancers is not always consistent.

The role of EMT transcription factors (EMT-TFs) in melanoma phenotype switching and plasticity has recently been reviewed (27). Induction of ZEB1 and SNAIL family members as discussed by Vandamme and Berx, as well as repression of E-cadherin is observed during melanoma progression. The traditional paradigm in epithelial cancers is that the EMT-TFs SNAIL1/2, ZEB1/2, and TWISTS act as repressors of E-cadherin, thereby inducing EMT (9). However, unlike epithelial cancers, in melanoma ZEB1 and ZEB2 are reported to be differentially expressed in alternate phenotypic states (28). Normal epidermal melanocytes from a melanoma patient expressed low ZEB1 and high ZEB2 expression, whereas the melanoma cells at deep sites from the same patient had high ZEB1 and low ZEB2 levels (28). Analysis of a large patient series by immunohistochemistry revealed high expression of ZEB1 and TWIST1, with low expression of ZEB2 corresponded with significantly reduced metastasis-free survival (28). Another recent study analyzing a large cohort of patient samples also confirmed that low expression of ZEB2 corresponded to significantly reduced melanoma recurrence-free survival (29). The study also demonstrated that loss of ZEB2 in melanocytes resulted in dedifferentiation, and in melanoma cells resulted in increased ZEB1expression, repressing E-Cadherin, and contributing to progression and metastasis (29). These studies suggest that ZEB2 could function as a differentiation factor, through maintaining E-Cadherin expression (29). Both studies also reported that the melanoma differentiation marker microphthalmia-associated transcription factor (MITF) was regulated by the switch in ZEB expression. Down-regulation of MITF could lead to an invasive phenotype, consistent with the previous literature on the role of MITF in phenotype switching (25, 27). Gene expression profiling comparing non-metastatic and metastatic patient samples, previously revealed that loss of E-cadherin/gain of N-cadherin was a major determinant of melanoma metastasis (24). The relevance of this cadherin switch was established in early studies on prostate and melanocytic cancers (30, 31), whereas SPARC was found later to drive activation and sustain expression of SLUG to promote melanoma cell invasion (32). SLUG was also identified in melanoma cell lines as a direct transcriptional activator of ZEB1, resulting in repression of E-cadherin (33). Interestingly in contrast, switching to a proliferative state was reported to occur in aggressive uveal melanoma with up-regulation of E-cadherin. However, the study revealed that this phenomenon was caused by the loss of an E-Cadherin suppressor called Id2, and as a result of down-regulation of Id2 there was increased anchorage-independent growth of the cells (34). These studies suggest that the interchange between epithelial-like and mesenchymal-like phenotypes is context dependent in different types of melanoma, but the ability to switch phenotype in various types of melanoma has been implicated in conferring a higher risk of death due to metastasis. The dynamic switch back and forth between proliferative and invasive states is the model that is biologically reflective of melanoma progression (35).

Phenotype switching in melanoma can be initiated by mechanisms other than those characterized in EMT. In epithelial cancer cell lines, increased LEF1 transcription activity by stable nuclear beta-catenin expression can induce EMT, which is reversible by removal of LEF1 (36). In melanoma, the beta-catenin interacting factors LEF1 and TCF4 are both expressed in a phenotype-specific manner and their expression is inversely correlated. Loss of LEF1 and gain of TCF4 expression is associated with tumor progression involving a change from proliferative to an invasive phenotype (37). The beta-catenin/LEF1 complex is regulated by Wnt signaling and activates a melanocyte-specific gene encoding MITF (38). MITF is a master regulator of melanocyte development and has been reported to be critical for melanoma progression (39, 40). MITF can control melanoma cell differentiation and proliferation through cell cycle arrest (41, 42). It also regulates diaphanous-related formin Dia1, which promotes actin polymerization and coordinates cytoskeletal networks at the cell periphery resulting in morphological changes (43). Expression of MITF has been used as a benchmark to distinguish melanoma cells in the proliferative or invasive state (25). In addition, Wnt activation, rather than acting via the classical Wnt pathway, can signal through the Protein Kinase C pathway to mediate an EMT-like phenotype switch and melanoma migration (44). These studies, as summarized in Table 1, indicate that EMT-like phenotype switching can be induced at both transcriptional level and through well-defined canonical signaling pathways.

**Table 1:** **Inducers of phenotype switching in melanoma**.

Phenotype switching inducers	Outcome	Study model^a^	Type of melanoma	Reference
↓ZEB2	↓Metastasis-free survival	Patient, *in vivo* and *in vitro*	multiple	(29)
↑ZEB1&TWIST/↓ZEB2&SLUG	↓Metastasis-free survival	Patient and *in vitro*	multiple	(28)
↑MITF	Differentiation	*In vitro* and *in vivo*	Cutaneous	(45)
EGF/STAT3	Growth and Metastasis	*In vitro* and *in vivo*	Cutaneous	(75)
WNT5A/↑ROR2	Invasion	*In vitro* and *in vivo*	Cutaneous	(46)
MET/Exosome	Metastasis	Patient and *in vivo*	Cutaneous	(47)
↑TCF4/↓LEF1	Invasion	*In vitro*	Cutaneous	(37)
↓MITF	Invasion	*In vivo*	Cutaneous	(25)
WNT5A/PKC	Migration	*In vitro*	Cutaneous	(44)
FGF2/↓FAK	Migration	*In vitro*	Cutaneous	(48)
↓E-Cad/↑N-Cad	Metastasis	Patient	Cutaneous	(24)
↑E-Cad	Invasion	*In vivo*	Uveal	(34)
HGF/Fibronectin	Migration	*In vitro*	Cutaneous	(49)
IGF-1	Migration	Patient and *in vitro*	Uveal	(50)

*^a^*In vitro* indicates melanoma cell lines in 2D culture, *in vivo* indicates xenograft models or mouse models and Patient indicates patient samples*.

## Signaling Pathways Involved in Melanoma Phenotype Switching

Wnt and Notch signaling have well-characterized roles in developmental programs of neural crest cells (51, 52). These embryonic signaling pathways are also implicated in tumorigenic functions of melanoma cells (53). Notably, melanoma have a high frequency of activating mutations within the MAPK pathway, as over 50% metastatic melanomas are driven by the oncogenic BRAF^V600E^ mutation (54) and over 15% by the NRAS^Q61R^ mutation (55). The MAPK and the PI3K signaling pathways are known to activate NF-kB, which further induces Snail to mediate a mesenchymal phenotype in epithelial cells (56), but similar evidence for the NF-kB/Snail mechanism in melanoma is lacking (57), although Snail is a demonstrated inducer of the mesenchymal-like phenotype in melanoma (58). However, this study may suggest that RTKs could be a means of mediating NF-kB/Snail activation given that they activate the MAPK and PI3K signaling pathways.

Additionally, there is abundant evidence that RTK signaling can induce migratory, invasive, and metastatic properties in melanoma cells. Knockdown of EGF in EGF over-expressing melanoma cells results in reduced lymph node metastasis, which is considered a key initial step of melanoma progression (59). FGF2 is a growth factor produced by melanoma cells but not by normal melanocytes, that activates the FGFR1 receptor. FGF2 promotes melanoma cell migration via down-regulation of focal adhesion kinase (FAK) and subsequent loss of cellular adhesion (48). As previously discussed, a cadherin switch is an important marker of EMT-like phenotype switching in melanoma. By studying exogenously introduced HGF ligand-induced activation of its receptor MET and pharmacological inhibition of downstream MAPK and AKT signaling, HGF signaling was shown to mediate the cadherin switch through up-regulation of Snail and Twist (60). Additionally, HGF signaling can also induce fibronectin matrix synthesis, which promotes malignant transformation and migration of melanoma cells (49). IGF-1 can also induce migration, through increased production of IL-8 by melanoma cells (61). In patients with uveal melanoma, a significant correlation was found between high expression of IGF-1 receptor and liver metastasis (50).

TGF-β is the most extensively studied inducer of EMT, with established roles in regulating extracellular matrix remodeling and in influencing cell phenotype (62, 63). Moreover, TGF-β can signal through SMAD3 and activate SNAI2/SLUG in a Rho-pathway dependent manner (64). Enhanced TGF-β signaling is implicated in mediating resistance to the inhibition of a range of oncogenic signaling targets. Loss of MED12, a repressor of TGF–βR2 signaling, not only confers a mesenchymal phenotype, but also results in resistance to inhibitors of ALK, EGFR, and BRAF in multiple cancers including melanoma (65).

## Implications of Phenotype Switching on Responses to Therapies

Uncontrolled proliferation is a cancer hallmark, a result of activation and crosstalk of many signaling pathways. Advances in genomic sequencing technology have enabled the successful identification of the key oncogenic events in melanoma, including identification of the BRAF^V600E^ mutation (54). Subsequent developments of highly selective and efficacious therapies such as vemurafenib and dabrafenib that target mutant BRAF have achieved remarkable responses in patients (66–68). However, ongoing clinical studies have revealed that the therapeutic benefits are often short-lived with the majority of patients developing resistance and disease progression (66). There are several reports on the mechanisms of resistance to BRAF directed agents as reviewed by Sullivan and Flaherty (69). Besides the intrinsically resistant clones, some of the surviving drug-sensitive melanoma cells are able to adapt to BRAF inhibition. Studies have revealed that the adaptation can involve various phenotype changes including EMT-like processes, altered glycolytic activity (70) and ER stress response-activated cytoprotective autophagy (71). Hypoxia induced switching of the expression of ROR1 and ROR2 through non-canonical WNT5A signaling, resulting in an invasive phenotype of melanoma with reduced sensitivity to BRAF inhibitors (46). Concurrent inhibition of BRAF and glycolysis or autophagy was demonstrated as good methods to induce cell death or tumor regression, respectively, in BRAFi-resistant melanoma (71, 72). However, to target phenotypic-switching through therapeutic intervention remains difficult. Thus, the remainder of this mini-review will emphasize the involvement of phenotype switching in the context of emerging and recently developed therapies.

Using BRAF^V600E^ melanoma lines and BRAF inhibitors, Caramel et al., demonstrated that the ZEB switch described above, can be initiated and sustained by MAPK/ERK signaling through FRA-1, an ERK-regulated component of the AP-1 complex. Accordingly, the expression patterns of ZEB1/2 and TWIST were reversed by pharmacological inhibition of BRAF/ERK signaling (28). Together with the TGF-β/MED12 study that showed changes of expression of phenotype markers concomitant with development of drug resistance (65), these recent discoveries support the emerging understanding that the mechanisms of phenotype switching in melanoma may have broader implications with respect to therapeutic responses in patients.

An important question raised by all the studies described above is whether EMT-like phenotype switching has any value as a therapeutic “target” in the treatment of melanoma. To date, three major strategies have been proposed to address this important question. Considering the aggressiveness of melanoma, the first suggested approach is to directly reduce invasive potential. Compounds such as the potent green tea catechin, Epigallocatechin gallate (EGCG), have been demonstrated to have inhibitory effects on migration and invasion in the BRAF-mutant cell line A375, with a reversal of EMT-like phenotypic changes orchestrated by induction of E-cadherin and suppression of N-cadherin (73). A second reported strategy is to use phenotype switching as a method to induce changes in melanoma to a specific phenotype that reveals a “drug-targetable” state. As previously discussed, high expression of MITF usually associates with a proliferative phenotype in melanoma. The chemotherapeutic agent methotrexate (MTX) causes an increase in MITF and its direct target TYR (tyrosinase) that inhibit invasiveness in melanoma. This can provide an avenue for treatment with a tyrosinase-processed antifolate pro-drug that was shown to mediate apoptosis selectively in the MTX-treated cells with high expression of MITF and tyrosinase (45). The third reported strategy is based on the success of the approved and emerging therapies targeting the BRAF/MAPK signaling in melanoma. Phenotype switching, cell migration, and invasion occur instead of, or concomitantly with, the development of drug resistance (65). Thus, the rationale involves inhibition of phenotype switching and cell migration in conjunction with a therapy such as vemurafenib that targets the oncogenic BRAF signaling that leads to growth arrest or/and cell death. Studies reveal that combination of inhibitors of TGFβR2 with vemurafenib overcomes the TGFβ-mediated resistance to vemurafenib (65). Chronic inhibition of BRAF was also found to result in elevated Wnt signaling and increased expression of the EMT inducer, WNT5A, and knockdown of WNT5A reversed resistance caused by chronic treatment with vemurafenib (74).

Given that signaling by various RTKs can mediate phenotype switching and promote migration through mechanisms distinct from those enhancing BRAF/MAPK-dependent proliferation and regulation of EMT-TFs, co-targeting of selected RTK signaling pathways and oncogenic BRAF appears to be a logical combination. For example, EGF signaling confers resistance to BRAF inhibition and induces melanoma invasion through Src pathways. Inhibition of the EGF receptor and Src re-sensitizes treatment-resistant BRAF-mutant melanoma cells to Vemurafenib and blocks their invasiveness (75). HGF secreted by stromal cells in the tumor microenvironment can activate the HGF receptor MET, initiating MAPK and PI3K signaling to confer resistance to BRAF inhibition. Consistently, dual inhibition of either HGF or MET was found to forestall the resistance (76). This may be of particular importance because melanoma-derived exosomes were able to confer metastatic properties and a pro-vasculogenic phenotype on bone marrow progenitors through MET (47). Exosomes are important export machinery that maintains normal compartmentalization of molecules. In a range of cancers including melanoma, exosomes derived from melanoma cells contain oncogenic drivers influencing EMT and metastasis (77). Interfering with regulators of exosome formation and MET expression can reduce metastasis (47).

## Conclusion

**Figure 1:**
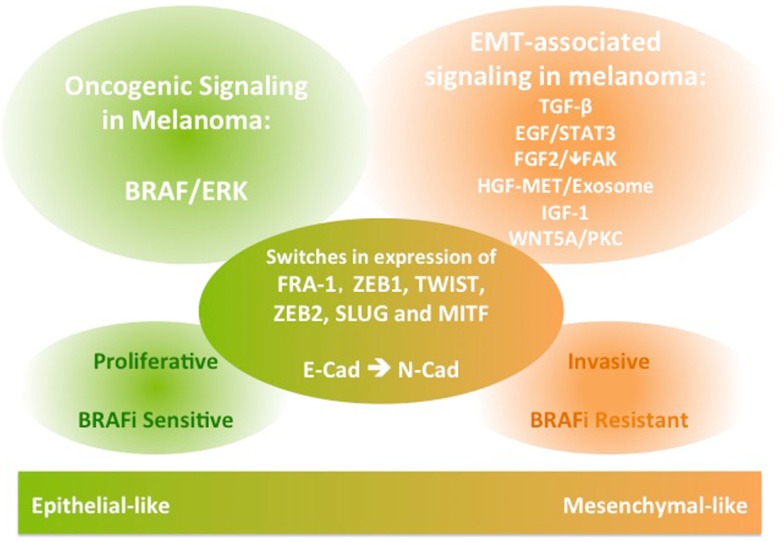
**A schematic diagram of the signaling and molecular features of melanoma phenotype switching**. The EMT-like phenotype switching confers melanoma invasive functions. The EMT-associated signaling in melanoma is also implicated in conferring resistance to BRAF inhibition therapies in BRAF-mutant metastatic melanoma.

The EMT process is crucial for normal development and for initiation of malignant transformation and metastasis in a wide range of epithelial cancers. It involves activation of various signaling pathways, as well as repression of E-cadherin through transcription factors. EMT-like phenotype switching is critical for melanocyte lineage differentiation and initiation of melanoma transformation and metastasis. While common EMT-TFs are implicated, their expression during the switch of melanoma to a mesenchymal-like invasive phenotype can differ from the role in classical EMT. In addition to TGFβ and WNT5A signaling, EGF, FGF, MET, and IGF signaling have established roles in conferring migratory and invasive functions in melanoma (Figure 1). Importantly, these EMT-associated signaling pathways also have roles in conferring resistance to inhibitors of BRAF/MEK, hindering therapeutic outcomes in patients with metastatic melanoma driven by BRAF mutations. Therefore, integrating insights from this body of literature may aid in the design of studies aiming to predict the patterns of melanoma progression during treatment with targeted therapeutics and may facilitate development of novel combination therapies.

## Author Contributions

FL wrote the mini-review. PF, AD, GM, and RA provided intellectual input and contributed to editing the manuscript.

## Conflict of Interest Statement

GM is on uncompensated advisory boards for GSK, Roche-Genentech, Novartis, BMS, Millenium, Merck and Amgen, is a consultant for Provectus and receives research grant support from Novartis, Pfizer, Ventana, and Celgene. The other authors declare no commercial or financial relationships that could be construed as a potential conflict of interest.

## Abbreviations

EGF, epidermal growth factor; EMT, epithelial–mesenchymal transition; EMT-TF, epithelial–mesenchymal transition-transcription factors; FGF, fibroblast growth factor; HGF, hepatocyte growth factor; IGF, insulin-like growth factor; KIT, stem cell factor receptor; MAPK, mitogen-activated protein kinases; MET, hepatocyte growth factor receptor; MITF, microphthalmia-associated transcription factor; PI3K, phosphatidylinositol-4,5-bisphosphate 3-kinase; ROR, receptor tyrosine kinase-like orphan receptor; RTK, receptor tyrosine kinases; STAT, signal transducer and activator of transcription; TGF, transforming growth factor.
